# Ionic Liquids as Homogeneous Catalysts for Glycerol Oligomerization

**DOI:** 10.3390/polym14061200

**Published:** 2022-03-16

**Authors:** Dawid Kansy, Krystyna Czaja, Kornelia Bosowska, Paweł Groch

**Affiliations:** Faculty of Chemistry, University of Opole, 45-052 Opole, Poland; czaja@uni.opole.pl (K.C.); kornelia.bosowska@uni.opole.pl (K.B.); pawel.groch@uni.opole.pl (P.G.)

**Keywords:** ionic liquids, glycerol, oligoglycerol, oligomerization, catalysis, polyglycerols, polymerization

## Abstract

Ionic liquids (ILs) were used for the first time as catalysts for the glycerin condensation reaction. A series of imidazolium and ammonium ionic liquids differing in the length of the alkyl substituent (C_2_, C_12_, and C_14_) and the type of anion (Br^−^, CH_3_COO^−^, and NaHPO_4_^−^) were synthesized using a typical two-step method. The structure of the obtained ILs was confirmed by nuclear magnetic resonance ^13^C NMR, and their base power was determined on the basis of the Hammett function. The oligomerization of glycerin with the participation of the obtained ionic liquids and, for comparison, in the presence of a homogeneous basic catalyst Na_2_CO_3_, was carried out for 3 h at 180 °C, under a pressure of 0.4 bar, where the highest conversion, i.e., 92%, was obtained against 1-dodecyl-*N*,*N*,*N*-triethylammonium acetate. The course of the reaction was monitored using a reaction system coupled with a FTIR spectrometer, which allowed for the tracking of changes in product concentration over time and the assessment of glycerin oligomerization kinetics. The reaction products were analyzed by positive electrospray ionization mass spectrometry (ESI-MS), ^13^C NMR, and infrared absorption spectroscopy (FTIR).

## 1. Introduction

The contemporary interest in polyglycerols (oligoglycerols) results from the need to manage a large amount of waste glycerin [1], which is mass produced in biofuel synthesis processes in the reaction of fat transesterification with methyl or ethyl alcohol [2,3,4]. As a result, new methods of synthesis are being developed, and the range of applications of oligoglycerols differing in the degree of oligomerization and composition is being extended.

The obtained oligoglycerols may have a linear [5], cyclic, or branched structure depending on the course of the glycerol condensation reaction [5], which may take place with the participation of a primary or secondary alcohol [5]. The factors that determine the obtaining of the desired oligoglycerol fraction are mainly the type and characteristics of the catalyst used and the reaction environment [5,6]. If the reaction is carried out in an alkaline environment, then the glycerin condensation proceeds according to the SN_2_ nucleophilic substitution reaction mechanism, whereas when acid catalysts are used, the reaction proceeds according to the SN_1_ nucleophilic substitution mechanism.

The condensation of glycerol is carried out using heterogeneous or homogeneous catalysts. Zeolites are mainly used as heterogeneous acid catalysts in the glycerol oligocondensation reaction. Their proton form shows a strong acid character, comparable even to strong mineral acids, thanks to the Brønsted acid centers formed by OH groups, which are bridging elements between the tetrahedral coordinated aluminum and silicon atoms in the crystalline aluminosilicates. For example, when converting glycerin using 20 g of Beta zeolite (Si/Al 50/50 molar ratio, 0.1% by weight of Na_2_O, grain size 0.1–0.7 µm, surface area 750 m^2^/g) in at 200 °C for 2 h, a product is obtained with 60% efficiency, which contains 30% linear diglycerol, 30% cyclic di-glycerol, 30% cyclic triglycerol, and 10% higher oligomers [6,7,8,9,10,11].

Conventional heterogeneous catalysts, however, have disadvantages such as the difficulty of reproducibility of their synthesis, and usually show lower activity compared to their homogeneous counterparts, which consequently requires the use of higher reaction temperatures and an extended reaction time.

The acidic homogeneous catalysts used so far are inorganic acids, which increase the reaction rate of the oligomerization process but deteriorate its selectivity [5]. In contrast, alkaline homogeneous catalysts, such as hydroxides, carbonates, or oxides of alkali metals, lead to products of various structures [5]. Our previous studies showed that, as a result of glycerin oligomerization carried out with the use of sodium carbonate as a basic homogeneous catalyst, it is possible to obtain a mixture of linear oligoglycerols with an average molecular weight exceeding even 400 g/mol containing an additional hydroxyl group at the α carbon atom [12]. The disadvantages of homogeneous catalysts, however, include their insufficient thermal stability and difficulties with their isolation from the post-reaction mixture, and at the same time they often cause corrosion of the equipment and generate the need to process a large amount of liquid waste.

In the last dozen or so years, ionic liquids include new, more effective, and less toxic catalysts. Additionally, they are characterized by quite good thermal stability, and it is possible to reuse them in subsequent reaction stages. Ionic liquids containing an imidazolium cation are used, inter alia, in in C–O bond opening reactions in epoxides, in alcohol etherification reactions with alkyl chloride in phase transfer catalysis (PTC), and in alcohol-alcohol etherification [13,14,15,16,17,18,19]. Moreover, the use of ammonium acetates in the etherification reactions of propylene oxide with alcohols leads to more selective products [15]. Taking the above into account, it seemed advisable to evaluate the possibility of using imidazolium and ammonium ionic liquids as homogeneous catalysts for glycerin oligomerization. It should be emphasized that there is no information in the literature on the use of ionic liquids as catalysts for the synthesis of oligoglycerols

## 2. Materials and Methods

### 2.1. Materials

Anhydrous glycerin (≥99.5%), pa M = 92.10 g/mol from Chempur (Piekary Slaskie, Poland); sodium carbonate, anhydrous (≥99.5%), pa M = 105.99 g/mol, acetic acid (≥99.5%), pa M = 60.05 g/mol, and sodium hydrogen phosphate (≥99.5%), pa M = 141.96 g/mol from POCH (Gliwice, Poland); and nitrogen (Messer, Chorzów, Poland) were purchased. In turn, 1-methylimidazole (≥99%), pa M = 82.10 g/mol, bromoethane (≥98%), pa M = 108.97 g/mol, and *N*,*N*-diethylethanamine (≥99%), pa M = 101.19 g/mol was purchased from Sigma Aldrich (Poznań, Poland), and a 1-bromotetradacane (≥98%), pa M = 277.28 g/mol and 1-bromododecane (≥98%), pa M = 249.23 g/mol in Acros Organics (Geel, Belgium); were purchased from POCH (Gliwice, Poland). All reagents were used without further purification.

### 2.2. Synthesis Ionic Liquids

**Figure 1:**
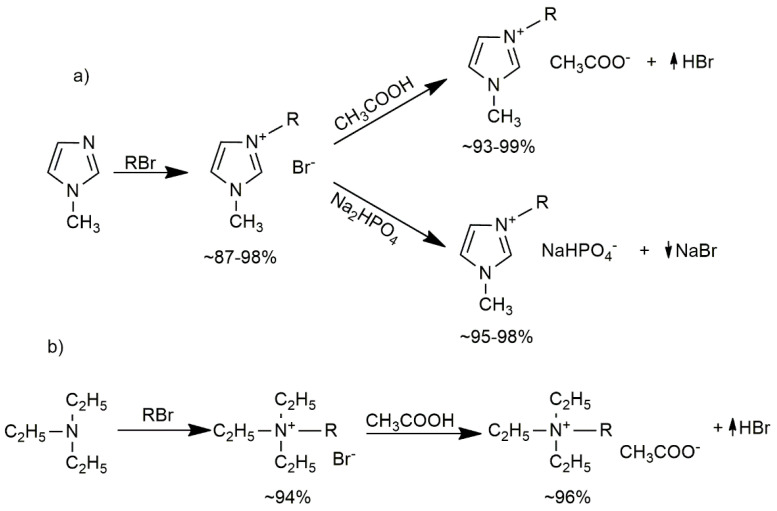
Two-step scheme for the synthesis of ILs: (**a**) ILs imidazoliums; (**b**) ILs ammonia, where R: group alkyl C_2_H_5_, C_12_H_25_, C_14_H_29_.

Ionic liquids used in this work were obtained by a typical two-step method involving quaternization of imidazole or amine followed by anion exchange [15]. The general scheme of the reaction is presented in Figure 1.

3-Alkyl-1-methylimidazolium bromides differing in the alkyl substituent (ethyl, dodecyl, and tetradecyl, respectively ([C_2_-mim][Br], [C_12_-mim][Br], and [C_14_-mim][Br]), were prepared by reacting 1-methylimidazole with the appropriate alkyl bromide (Figure 1a). The synthesis was carried out in a three-neck round bottom flask with a capacity of 250 mL, into which 1-methylimidazole and 100 mL of toluene were introduced. The contents of the flask were heated to a temperature of 110 °C (for [C_2_-mim][Br]) or 70 °C (for [C_12_-mim][Br] and [C_14_-mim][Br]), then the appropriate alkyl bromide in an amount of 1.2 moles per mole of imidazole was slowly added dropwise. After its complete introduction, the synthesis reaction of the appropriate ionic liquid was carried out for 4 h or 5 h (in the case of [C_14_-mim][Br]). The solvent was then removed from the reaction mixture using a rotary evaporator. For purification, the obtained crude product was washed several times with 50 mL of a suitable solvent (ethyl acetate for [C_2_-mim][Br], toluene for the rest). The purified 3-alkyl-1-methylimidazolium bromide was then weighed to determine the synthesis yield. The obtained imidazolium bromides obtained with a purity of 98% were analyzed by nuclear magnetic resonance spectroscopy to confirm their structures:

[C_2_-mim][Br]: ^1^H NMR (DMSO-d_6_): 1.64 (t, 3H, CH_3_–CH_2_), 2.88 (s, 1H, N=CH–N), 3.40 (s, 3H, CH_3_–N), 4.79 (d, 2H, CH=CH), 5.07 (m, 2H, CH_2_–N); ^13^C NMR (DMSO-d_6_): 13.32 (CH_3_–CH_2_), 34.18 (CH_3_–N), 34.52 (CH_2_–N), 120.85–123.96 (CH=CH), 138.73 (N=CH–N).

[C_12_-mim][Br]: ^1^H NMR (DMSO-d_6_): 0.98 (t, 3H, CH_3_–CH_2_), 1.30–1.37 (m, 18H, CH_2_), 1.92 (m, 2H, CH_2_–CH_2_–CH_2_), 3.19 (s, 1H, N=CH–N), 3.35 (s, 3H, CH_3_-N), 4.62 (m, 2H, CH_2_-N), 4.91 (m, 2H, CH=CH); ^13^C NMR (DMSO-d_6_): 13.37 (CH_3_–CH_2_), 22.12–29.75 (CH_2_), 31.35 (CH_3_–N), 49.64 (CH_2_-N), 121.14–122.89 (CH=CH), 137.03 (N=CH–N).

[C_14_-mim][Br]: ^1^H NMR (DMSO-d_6_): 0.99 (t, 3H, CH_3_–CH_2_), 1.33 (m, 22H, CH_2_), 1.94 (m, 2H, CH_2_–CH_2_–CH_2_), 3.07 (s, 1H, N=CH-N), 3.42 (s, 3H, CH_3_–N), 4.62 (m, 2H, CH_2_–N), 4.88–4.93 (m, 2H, CH=CH); ^13^C NMR (DMSO-d_6_): 13.58 (CH_3_–CH_2_), 22.13–32.37 (CH_2_), 33.59 (CH_3_–N), 49.66 (CH_2_–N), 121.06–122.77 (CH=CH), 137.13 (N=CH-N).

The synthesis of 1-dodecyl-*N*,*N*,*N*-triethylammonium bromide [C_12_-tea][Br] was done in a similar way as for the synthesis of bromides 3-alkyl-1-methylimidazoliums using *N*,*N*,*N*-triethylamine in place of imidazole and an equimolar amount of the reactants (Figure 1b). The reaction was carried out at 60 °C for 4 h. After evaporation of the solvent, the obtained crude product was washed several times with 50 mL of acetonitrile, obtaining a yellow waxy solid [C_12_-tea][Br] with a yield of 94%.

[C_12_-tea][Br]: ^1^H NMR (DMSO-d_6_): 1.01 (m, 3H, CH_2_–CH_3_), 1.30–1.36 (m, 27Hoverlap signals for CH_3_ groups come from the ethyl substituent and CH_2_ from the C_12_ alkyl chain), 1.80 (t, 2H, N–CH_2_–CH_2_), 3.41 (t, 2H, N–CH_2_–CH_2_), 3.769–3.72 (q, 6H, N–CH_2_–CH_3_); ^13^C NMR (DMSO-d_6_): 10.02 (CH_3_–CH_2_–N), 14.02 (CH_3_–CH_2_–), 24.39–31.65 (CH_2_), 50.41 (CH_3_–CH_2_–N), 54.07 (N–CH_2_–CH_2_–).

In the second stage of the anion exchange reaction, the corresponding imidazolium or ammonium acetates were obtained according to Figure 1. The anion exchange in the imidazolium liquid was carried out in an Erlenmeyer flask placed on a magnetic stirrer with a heating function, to which the appropriate 3-alkyl-1-methylimidazolium bromide was introduced, dissolved in 50 mL of H_2_O, and then added, in excess (2:1), of 99.5% acetic acid. The system was heated to 40 °C to completely dissolve the bromide, then the reaction was carried out at 25 °C with continuous stirring for 24 h for [C_2_-mim][OAc] or 72 h for the synthesis of [C_12_-mim][OAc] and [C_14_-mim][OAc]. After completion of the reaction, the solvent was evaporated on a rotary evaporator at a temperature of 90 °C, and the obtained solid product was washed several times with distilled water, which was evaporated on a rotary evaporator. A quantity of 20 mL of iso-propanol was then added to dry and purify the product, and the solvent was re-evaporated on a rotary evaporator at 90 °C.

[C_2_-mim][OAc]: ^1^H NMR (DMSO-d_6_): 1.64 (t, 3H, CH_3_-CH_2_), 2.39 (m, 3H, CH_3_–COO), 3.23 (s, 1H, N=CH–N), 3.40 (s, 3H, CH_3_-N), 4.71–4.75 (d, 2H, CH=CH), 5.07 (m, 2H, CH_3_–CH_2_–N); ^13^C NMR (DMSO-d_6_): 13.32 (CH_3_–CH_2_), 22.65 (CH_3_–COO), 34.18 (CH_3_-N), 34.52 (CH_2_–N), 120.85–123.96 (CH=CH), 138.73 (N=CH-N),169.08 (CH_3_–COO).

[C_12_-mim][OAc]: ^1^H NMR (DMSO-d_6_): 0.99 (t, 3H, CH_3_–CH_2_), 1.30–1.37 (m, 18H, CH_2_), 1.95 (m, 2H, CH_2_–CH_2_–CH_2_), 2.39 (m, 3H, CH_3_–COO), 3.24 (s, 1H, N=CH–N), 3.33 (s, 3H, CH_3_–N), 4.62 (m, 2H, CH_2_–N), 4.91 (m, 2H, CH=CH); ^13^C NMR (DMSO-d_6_): 13.52 (CH_3_–CH_2_), 22.06 (CH_3_-COO), 22.64–31.27 (CH_2_), 36.14 (CH_3_–N), 49.51 (CH_2_–N), 121.41–123.18 (CH=CH), 136.57 (s, 1H, N=CH–N), 173.61 (CH_3_–COO).

[C_14_-mim][OAc]: ^1^H NMR (DMSO-d_6_): 0.99 (t, 3H, CH_3_-CH_2_), 1.31 (m, 22H, CH_2_), 1.95 (m, 2H, CH_2_–CH_2_–CH_2_), 2.39 (m, 3H, CH_3_–COO), 3.24 (s, 1H, N=CH–N), 3.33 (s, 3H, CH_3_–N), 4.62 (m, 2H, CH_2_–N), 4.71–4.76 (m, 2H, CH=CH); ^13^C NMR (DMSO-d_6_): 13.58 (CH_3_-CH_2_), 22.65 (CH_3_-COO), 28.29–29.37 (CH_2_), 33.59 (CH_3_-N), 49.66 (CH_2_-N), 121.06–123.77 (CH=CH), 137.13 (N=CH–N), 169.08 (CH_3_–COO).

Anion exchange was performed in a similar manner in 1-dodecyl-*N*,*N*,*N*,-triethylammonium bromide and reacted at 25 °C for 72 h.

[C_12_-tea][OAc]: ^1^H NMR (DMSO-d_6_): 0.99 (m, 3H, CH_2_-CH_3_), 1.22–1.37 (m, 27H, overlap signals for CH_3_ groups come from the ethyl substituent and CH_2_ from the C_12_ alkyl chain), 1.78 (m, 2H, N–CH_2_–CH_2_), 2.41 (m, 3H, CH_3_–COO–), 3.41 (m, 2H, N–CH_2_–CH_2_), 3.84 (m, 6H, N–CH_2_–CH_3_); ^13^C NMR (DMSO-d_6_): 10.02 (CH_3_–CH_2_–N), 14.02 (CH_3_–CH_2_-), 28.25 (CH_3_–COO), 24.39–31.85 (CH_2_), 50.41 (CH_3_–CH_2_–N), 54.07 (N–CH_2_–CH_2_–), 169.08 (CH_3_–COO).

The synthesis of sodium hydrogen phosphate of 3-dodecyl-1-methylimidazole was carried out by anion exchange in the appropriate imidazolium bromide ([C_12_-mim][Br] and [C_14_-mim][Br]) to the sodium hydrogen phosphate anion (Figure 1a, second step). The appropriate bromide was dissolved in water and an equimolar amount of Na_2_HPO_4_ was added. The exchange reaction was carried out at 25 °C for 24 h with constant stirring. The solvent was then evaporated on a rotary evaporator at 90 °C and the product was washed several times with ethanol. The precipitated sodium bromide was filtered off, and the corresponding ILs was isolated from the remaining solution after evaporation of the solvent. The NMR analysis results were as follows:

[C_12_-mim][NaHPO_4_]: ^1^H NMR (DMSO-d_6_): 0.99 (t, 3H, CH_3_-CH_2_), 1.30–1.37 (m, 18H, CH_2_), 1.95 (m, 2H, CH_2_–CH_2_–CH_2_), 2.39 (m, 3H, CH_3_–COO), 3.24 (s, 1H, N=CH–N), 3.33 (s, 3H, CH_3_–N), 4.62 (m, 2H, CH_2_–N), 4.91 (m, 2H, CH=CH); ^13^C NMR (DMSO-d_6_): 13.52 (CH_3_-CH_2_), 22.06 (CH_3_–COO), 22.64–31.27 (CH_2_), 36.14 (CH_3_–N), 49.51 (CH_2_–N), 121.41–123.18 (CH=CH), 136.57 (s, 1H, N=CH-N), 173.61 (CH_3_–COO).

[C_14_-mim][NaHPO_4_]: ^1^H NMR (DMSO-d_6_): 0.99 (t, 3H, CH_3_-CH_2_), 1.37 (m, 22H, CH_2_), 1.95 (m, 2H, CH_2_–CH_2_–CH_2_), 2.39 (m, 3H, CH_3_–COO), 2.89 (s, 1H, N=CH–N), 3.39 (s, 3H, CH_3_-N), 4.62 (m, 2H, CH_2_-N), 4.71–4.76 (m, 2H, CH=CH); ^13^C NMR (DMSO-d_6_): 13.58 (CH_3_–CH_2_), 22.65 (CH3–COO), 28.29–29.37 (CH_2_), 33.59 (CH_3_-N), 49.66 (CH_2_-N), 121.06–123.77 (CH=CH), 137.13 (N=CH-N), 169.08 (CH_3_-COO).

### 2.3. Synthesis Oligoglycerols

The glycerin oligomerization process was carried out with the participation of ionic liquids and with sodium carbonate as the reference catalyst in the Mettler Toledo Easy Max 102 reactor, consisting of a five-necked 100 mL flask equipped with a thermocouple, a nitrogen bubbler attachment, the Mettler Toledo FTIR ReactIR15 probe, and a condenser for distillation under reduced pressure. The reaction was carried out using each IL in an amount of 1 wt.%. A quantity of 60 mL of glycerin in which an appropriate ionic liquid had previously been dissolved was introduced into the flask. The mixture was heated to 55 °C and nitrogen was bubbled through for 15 min to deoxygenate the system. The nitrogen flow was then stopped to prevent removal of the substrate from the reaction zone as the contents of the reactor were further heated. The condensation reaction was carried out under constant stirring with a stirrer with a magnetic dipole for 3 h at 180 °C and a pressure of 400 mbar. At this time, the reaction mixture was cooled to room temperature, and the obtained product was analyzed.

Equipping the reactor with a FTIR probe made it possible to track the kinetics of the glycerin oligomerization reactions carried out with the participation of ionic liquids. The course of the reaction was followed by the current analysis of the intensity changes of the band of asymmetric stretching vibrations of the C-O-C groups at the wavenumber of 1100 cm^−1^.

### 2.4. Analysis

Structure of ILs were analyzed on the basis of proton and carbon nuclear magnetic resonance—NMR spectra. Spectra were recorded on a Bruker Ultrashield 400 MHz spectrometer from Rheinstetten, Germany. For the proton spectra, the generation frequency was 400 MHz, and for the carbon spectra it was 100 MHz.

Using the JASCO 650 UV-Vis spectrophotometer from Pfungstadt, Germany, the Hammett function was determined by assessing the deprotonation degree of the bromothymol blue index (HI) in relation to the deprotonated form concentration [I^−^]. The Hammett function can be determined in the case of the dissociation of an ionic liquid in a specific solvent, where it is defined on the basis of the relationship H_0_ = pK (HI)_al._ + log([I^−^]_s_/[HI]_s_); where: pK (HI)_al._—pKa value of the indicator in the alcoholic solution, which was methanol. The indicator in the form of bromothymol blue (BTB) was dissolved in methanol in a concentration of 1.6 × 10^−5^ mol/dm^3^, whereas solutions of ionic liquids were prepared in a concentration of 0.01 mol/dm^3^. The highest absorbance of BTB in the unrotonated form was determined against 0.1 molar and 0.01 molar sodium hydroxide at a wavelength of 620 nm [14].

The melting points of the ILs were determined by the differential scanning calorimetry (DSC) method using a Mettler Toledo Differential Thermoanalyzer (Columbus, OH, USA) in a nitrogen atmosphere with a flow of 40 mL/min at a temperature increase of 10 °C/min, and their stability was assessed by the thermogravimetric method using a Mettler Toledo TGA 2050 analyzer. The analysis was carried out in the temperature range from 30 to 190 °C under a nitrogen atmosphere with a flow of (40 mL/min) with a temperature increase rate of 10 °C/min.

Glycerin oligomerization products were analyzed using the following techniques: mass spectrometry (MS) ESI-Q-TOF maXis impact instrument from Bruker Daltonik GmbH Germany; nuclear magnetic resonance (^13^C NMR) 100 MHz Ultrashield Bruker, instrument from Rheinstetten, Germany; and FTIR ReactIR 15 probes from Mettler Toledo. ESI-MS analyses were performed with an HR-ESI-MS system (Bruker Daltonics, Bremen, Germany) equipped with an electrospray (ESI) source. Mass spectra were recorded in the range 50–1300 *m*/*z* in the positive ionization mode. The analyzed samples were diluted with methanol to a concentration of 2 mg/mL and sonicated for 5 min, then 10 µL was taken and dissolved in 990 µL acetonitrile/water (50:50 *v*/*v*) with the addition of 0.1% formic acid, obtaining 100 times dilution. The mixture was directly added to the ESI source at a flow rate of 3 µL min-1 by means of a micro-syringe pump. High purity nitrogen was used as a drying and nebulizing gas. The following ESI source conditions were used: capillary temperature 180 °C, shielding gas flow rate 3L min^−1^, spray voltage −4000 V.

The rheological tests of the obtained oligoglycerols were carried out using the Discovery Hybrid Rheometer HR20 from TA Instruments. This rheometer is equipped with a furnace that enables the control of the measurement temperature and a plate-plate measurement geometry with a diameter of 25 mm; the gap was set to a height of 1 mm. The samples were applied directly between the plates and rheological analyses were performed at 30 °C. The linear viscoelasticity area of each sample was determined by deforming the sample with a constant frequency of 10 Hz determining the complex viscosity |η*| as a function of oscillation amplitude (γ) according to ASTM D7175 and DIN 51810-2. Measurement of the samples was carried out under isothermal conditions after their previous 10-min thermostation.

## 3. Results and Discussion

### 3.1. Characterization of ILs

**Figure 2:**
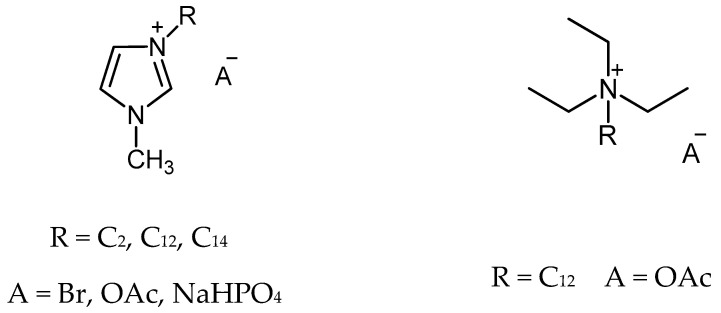
Ionic liquids used in study.

The imidazolium and ammonium ionic liquids with [Br], [CH_3_COO], and [NaHPO_4_] anions were obtained and tested. The selected ionic liquids also differed in the length of the alkyl substituent (C_2_, C_12_, C_14_) at the tertiary nitrogen atom of the imidazolium and ammonium cations (Figure 2).

Based on the evaluation of the efficiency of ILs synthesis, it was found that the exchange of the bromide anion for the acetate anion is definitely more effective compared to the exchange of the sodium orthophosphate anion. In the former situation, the exchange efficiency was always higher than 95% and even exceeded 99% during the synthesis of [C_2_-mim][OAc] and [C_12_-mim][OAc]. Slightly lower (81%) was the efficiency of the exchange of bromide into the acetate anion in the liquid with the 1-dodecyl-*N*,*N*,*N*-triethylammonium cation. In contrast, the appropriate exchange for the phosphate anion did not reach even 50%. This may be due to a steric hindrance due to the size differences of the two anions, further limited by the presence of large alkyl substituents in the cation.

All ionic liquids, regardless of the structure of the cation and anion, under ambient conditions were solid, colorless bodies ([C_2_-mim][Br] and [C_2_-mim][OAc]), which, along with the elongation of the alkyl substituents in the cation (C_12_ and C_14_), took a white color and even yellow in the case of [C_12_-tea][Br]. The melting points of the obtained ILs, determined by differential scanning calorimetry (DSC), were in the range 48–75 °C. The bromide [C_2_-mim][Br] (75.1 °C) was distinguished by clearly the highest value of the melting point. The remaining imidazolium bromides with C_12_ and C_14_ long alkyl substituents melted at a significantly lower temperature (53.5 and 55 °C, respectively). In contrast, after the exchange of the bromide anion, all liquids with the acetate or phosphate anion melted in a significantly lower temperature range (from 47.9 to 59.6 °C) than the starting bromides. Furthermore, 1-dodecyl-*N*,*N*,*N*-triethylammonium acetate, like the corresponding bromide, showed a melting point slightly higher (59.6 and 62.0 °C) than the melting range of similar liquids with the imidazolium cation containing the same alkyl substituent (54.5 and 53.5 °C).

Taking into account the planned use of the obtained ILs as catalysts for glycerin oligomerization carried out at 180 °C, their stability was tested under these conditions by heating them in a nitrogen atmosphere to 190 °C. On the basis of the recorded TGA thermograms, there was only a slight loss of sample mass, from 4 to 8%, in this range, which was probably caused by the evaporation of the remaining organic solvents and water.

Additionally, the key parameter of catalysts in many processes are their acid-base properties, as they directly affect the speed, selectivity, and efficiency of chemical reactions. Thus, the Hammett function was used to estimate the base strength of the obtained ionic liquids. Table 1 summarizes the Hammett function values calculated on the basis of the measured ratio [HI]/[I^−^], where [HI] is the concentration of the proton form and [I^−^] is the concentration of the deprotonated form. Bromothymol blue (BTB) was chosen as an indicator, and methanol was used as a solvent using prepared solutions of ionic liquids in this solvent at a concentration of 0.01 mol/dm^3^.

The results summarized in Table 1 show that all ionic liquids have pronounced alkaline properties. Additionally, they can be ranked from strongest to weakest rules in order: [C_14_-mim][OAc] > [C_14_-mim][NaHPO_4_] > [C_12_-mim][OAc] > [C_12_-tea][OAc] > [C_12_-mim][NaHPO_4_] > [C_2_-mim][OAc] > [C_2_-mim][Br]. As can be seen, imidazolium bromide showed the lowest basicity compared to the equivalents containing the acetate or phosphate anion, with the acetates showing the higher base strength of the latter. However, cation structure plays a key role in the basic nature of ILs. In the example of imidazolium liquids, regardless of the type of anion, their basicity increases with the increase of the length of the alkyl substituent. [C_14_-mim][OAc] acetate turned out to be the strongest base among all tested liquids, characterized by the value H_0_ = 12.92. The ionic liquid [C_14_-mim][NaHPO_4_] containing the imidazolium cation with the same alkyl substituent (C_14_) has a similar base strength (H_0_ = 12.88). Only slightly lower H_0_ values were found for imidazolium liquids (acetates and phosphates) with a slightly shorter dodecyl substituent (H_0_ 12.72 and 12.43, respectively). In contrast, significantly lower values of H_0_ = 11.21 and 11.74 showed imidazolium bromide and acetate, containing a much shorter ethyl substituent.

The observed differences in the values of the Hammett function of the analyzed ionic liquids show the influence of both the anion type and the cation structure on the basic strength of the liquid, which is the effect of the cation-anion interaction energy [15] and hydrogen-bond donor ability [20,21] and, consequently, the ability to dissociate a given liquid in solution.

### 3.2. Glycerin Oligomerization

The obtained ionic liquids were used as homogeneous catalysts for glycerol oligomerization, and their effectiveness was compared with the activity of a typical, homogeneous Na_2_CO_3_ basic catalyst. It should be emphasized that there is no information in the literature on the use of ionic liquids as catalysts for this reaction. Therefore, an important element of the work was to determine the influence of the structure of selected ionic liquids on the activity and selectivity of the glycerol oligomerization reaction.

**Figure 3:**
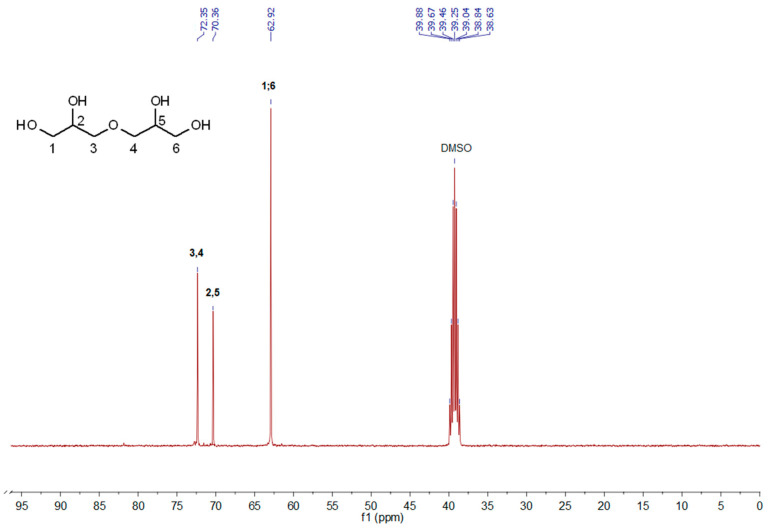
^13^C NMR (100 MHz, DMSO-d_6_) of oligomerization product obtained by [C_2_-mim][OAc].

The ^13^C NMR analysis of the products obtained with the tested ionic liquids confirmed the effectiveness of the syntheses carried out. In the exemplary ^13^C NMR spectrum (Figure 3), signals appearing at chemical shifts of 62.92; 70.36; and 72.35 ppm can be assigned to carbon atoms in the following groups: CH_2_–OH; CH_2_–CH(OH)–CH_2_; or CH_2_–O.

**Figure 4:**
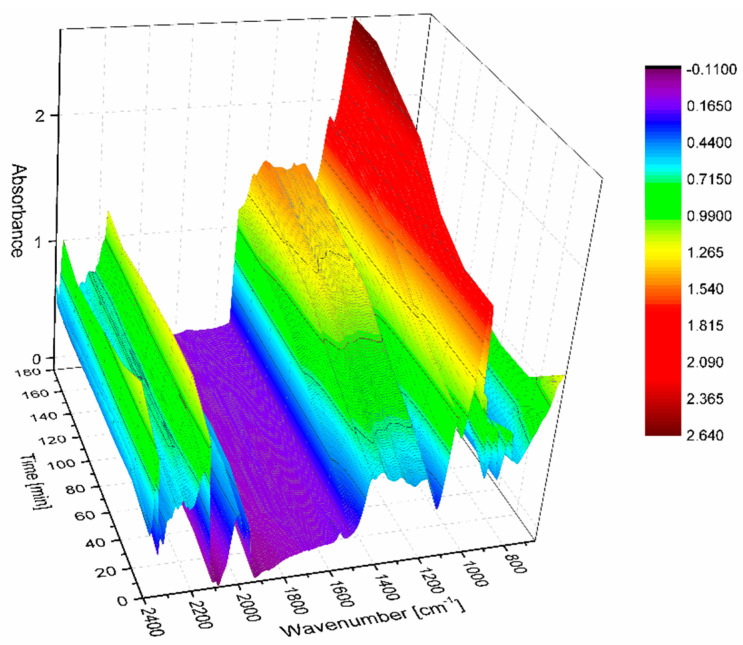
On-line monitored reaction spectra for glycerol oligomerization with [C_12_-mim][OAc].

During the glycerin oligomerization reaction, its progress was monitored by in-situ recording of the FTIR spectra of the reaction mixture. The set of spectra obtained over time of glycerol oligomerization catalyzed by the ionic liquid [C_12_-mim][OAc] is shown in Figure 4.

**Figure 5:**
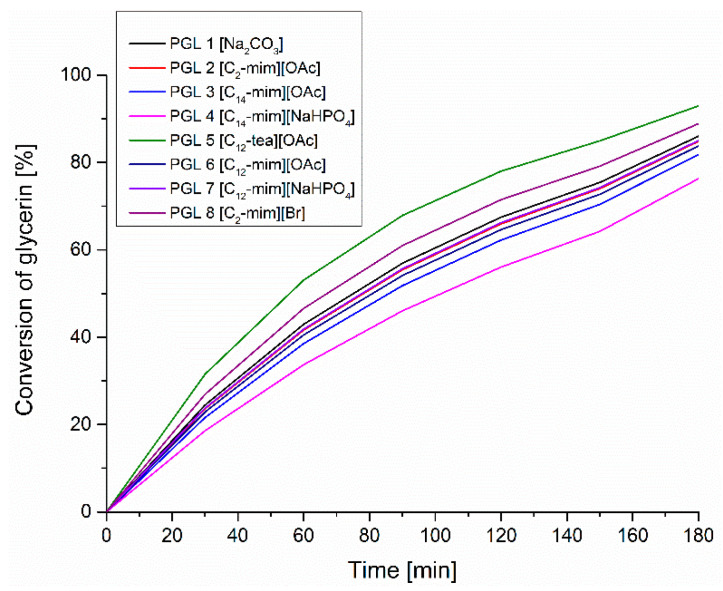
Dependence of glycerol conversion on reaction time determined on the basis of changes in absorbance of the C–O–C band at 1100 cm^−1^ recorded in-situ with the FTIR probe.

As can be seen, along with the course of the reaction, the band resulting from the vibrations stretching the C–O–C groups at 1100 cm^−1^ clearly grows, indicating the progress of the oligomerization reaction. The changes in the intensity of this band in relation to its absorbance at time t = 0, corresponding to the starting concentration of glycerin, were used to calculate the glycerin conversion as a function of time (Figure 5).

The measured values of the intensity of the band corresponding to the ether group as a function of time also allowed us to assess the rate of glycerol oligomerization reaction catalyzed with ionic liquids of different structures compared to the catalytic effect of sodium carbonate.

Considering that the reactivity of functional groups does not depend on the molecular weight of the substrate, and the reversible process can be omitted, because the equilibrium of the reaction was shifted to the right, due to the continuous reception of a by-product, which was water, the reaction rate constant can be determined from the pseudo-reaction rate equation primary (Equation (1)).

(1) $$\frac{dx}{dt}=\frac{-dx\left(c-x\right)}{dt}=k\left(c-x\right)$$

where: *c*—starting glycerin concentration, *x*—glycerin concentration over time *t*.

After separating the variables and taking the logarithm, the following relationship is obtained:

(2) $$\ln\left(\frac{c}{\left(c-x\right)}\right)=kt$$

which after transformation takes the form:

(3) $$k=\frac{1}{t}\ln\frac{c}{\left(c-x\right)}$$

Taking into account the measured values of the absorbance of the characteristic ether band in the course of the conducted experiments, Equation (3) takes the form:

(4) $$k=\frac{1}{t}\ln\left[\frac{\left(c_{0}-c_{k}\right)}{\left(c_{t}-c_{k}\right)}\right]$$

where: *c*_0_—the initial absorbance of the C–O–C band at 1100 cm^−1^,

*c_t_*—absorbance of this band over time *t*,

*c_k_*—the absorbance value of this band after the reaction is completed.

**Figure 6:**
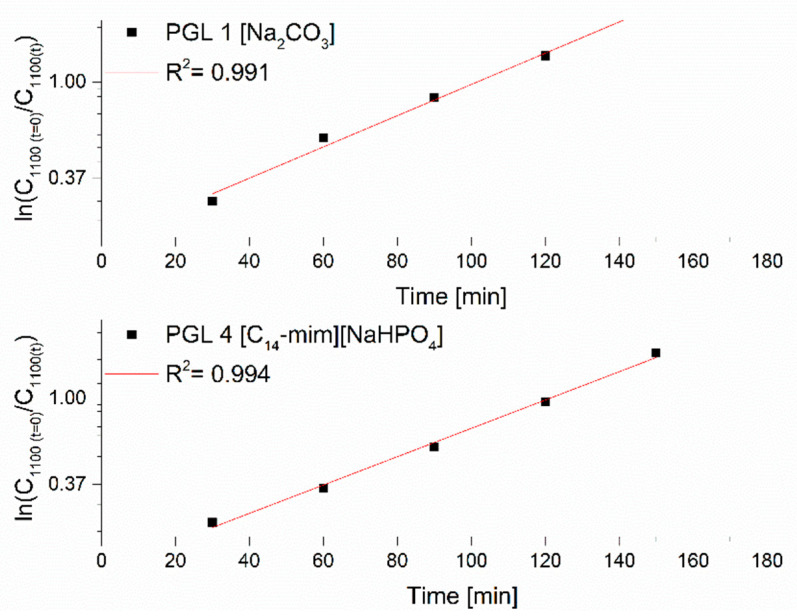
Consistent with Equation (4): Relationship of the absorbance of the C–O–C band at 1100 cm^−1^ as a function of time for the oligomerization of PGL 1 versus Na_2_CO_3_ and PGL 4 versus [C_14_-mim] [NaHPO_4_].

Using Equation (4), plots are plotted in a semi-logarithmic system of the corresponding absorbance ratio of the C–O–C ether group stretching band at 1100 cm^−1^ wavenumber as a function of time for a sodium carbonate catalyzed oligomerization reaction and an exemplary ionic liquid [C_14_-mim][NaHPO_4_]. As can be seen, rectilinear relations were obtained, with the correlation coefficient R^2^ > 0.9 (Figure 6).

The directional coefficients of the obtained rectilinear relations made it possible to determine the constant rate of the glycerol oligomerization reaction with the participation of various catalysts assessed here. Table 2 summarizes the results of calculations of the rate constant of the glycerin oligomerization reaction *k* along with the accuracy of its determination (R^2^), the final glycerin conversion (after 3 h) depending on the type of catalyst, and its basicity (at 0.01 mol%).

The data summarized in Table 2 show that among all the ionic liquids tested, the highest catalytic efficiency, expressed as the rate constant of the tested oligomerization reaction, equal to 2.10 × 10^−4^ s^−1^, and the highest conversion (92%), are characteristic of the ionic liquid [C_12_-tea][OAc], showing basicity expressed by the Hammett coefficient H_0_ = 12.64. Slightly lower activity (k = 1.74 × 10^−4^ s^−1^) was found for imidazolium bromide, although its basicity expressed by the Hammett function is the lowest (H_0_ = 11.21). All the remaining ionic liquids turned out to be even weaker catalysts, because the rate constant of the glycerin condensation reaction with their participation ranged from 1.14 × 10^−4^ s^−1^ to 1.51 × 10^−4^ s^−1^, and the conversion of the substrate after 3 h of reaction did not exceed 85%. At the same time, these liquids were mostly (except for [C_12_-mim][NaHPO_4_]) more basic (higher H_0_ value) than the most catalytically active liquid [C_12_-tea][OAc]. The obtained results show that the basicity of the ionic liquids is not crucial, and at least not the only parameter determining the speed of the glycerin oligomerization reaction. As can be seen, the rate of the reaction under study clearly decreases with the increase in the size of both the cation and the anion. The most active turned out to be the ionic liquid containing the ammonium cation, as well as liquids with an ethyl substituent on the imidazolium ring: ([C_2_-mim][OAc] and [C_2_-mim][Br]). Moreover, as the size of the substituent in the imidazolium cation increases, the rate of glycerol conversion also decreases ([C_2_-mim][OAc] > [C_12_-mim][OAc] > [C_14_-mim][OAc] or [C_12_-mim][NaHPO_4_] > C_14_-mim][NaHPO_4_]). These data indicate the negative role of steric hindrance, especially large alkyl substituents contained in the cation, limiting the rate of the reaction under study.

Subsequently, the composition of the obtained products (purified from catalyst residues by extraction with chloroform) was examined by analyzing them by means of electrospray mass spectrometry in positive ionization (ESI-MS). The aforementioned method was used earlier to determine the composition of the oligoglycerol mixture [18,19].

**Figure 7:**
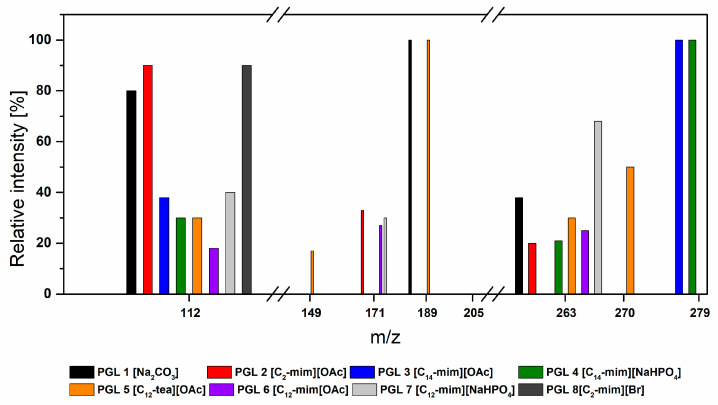
Influence of the catalyst type on the mass composition of the obtained oligoglycerols, determined on the basis of the ESI-MS spectra of the analyzed samples (*m*/*z* = [M^−^H]^+^).

In the ESI-MS spectra (Figure 7) of all the obtained products of the studied glycerol oligomerization reaction, the presence of several molecular peaks as well as the sodium, potassium, and ammonium adducts at different *m*/*z* values were found. These results show that regardless of the type of catalyst used, mixtures of oligoglycerols differing in structure and molecular weight were obtained. Moreover, in each post-reaction mixture, the presence of unreacted glycerin in the form of an adduct with the NH_4_^+^ cation was found, which proves incomplete conversion of this raw material, confirming the conversion results presented in Table 2.

**Figure 8:**
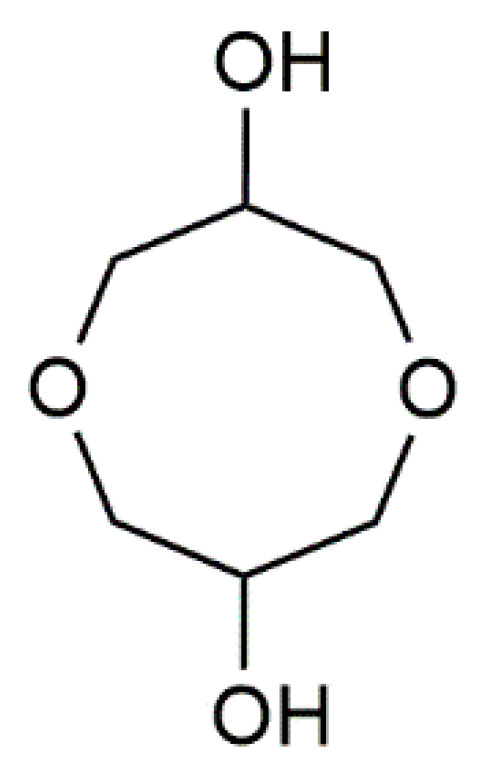
Structure of α, α-cyclodiglycerol.

The analysis of the spectra of the glycerol oligomerization products obtained both with the catalytic participation of ILs and with the reference Na_2_CO_3_ catalyst confirmed that they mainly contain two products with masses corresponding to triglycerol diglycerol adducts. A special exception was the mixture of oligoglycerols obtained from 1-dodecyl-*N*,*N*,*N*-triethylammonium acetate (PGL 5), in which, in addition to unreacted glycerin and the above-mentioned two linear, low-molecular-weight oligoglycerols, the presence of α, α-cyclodiglycerol was also found, with a mass *m*/*z* = 149 g/mol (Figure 8).

As it results from the data cited in Table 2, the glycerin oligomerization reaction with the participation of the said catalyst ([C_12_-tea][OAc]) stands out among the others with the highest reaction rate constant, and the effect is the highest glycerin conversion. The formation of the cyclic product can thus be caused by the rapid electrophilic nucleophilic activation and the steric hindrance caused by a long alkyl substituent in the catalyst cation, whereby glycerol molecules tend to form cyclic products. Interestingly, also in the post-reaction mixtures of glycerol oligomerization with the participation of ionic liquids containing the same dodecyl substituent in the imidazole ring, regardless of the anion type ([C_12_-mim][OAc], [C_12_-mim][NaHPO_4_]), it was also found presence of α, α-cyclodiglycerol in the form of an adduct with the sodium cation (*m*/*z* = 171 g/mol).

**Figure 9:**
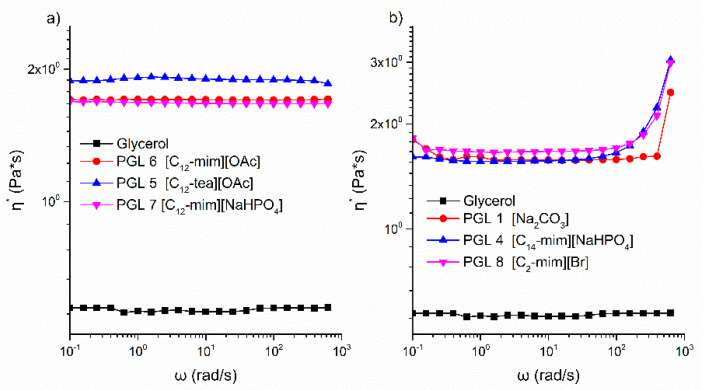
Graph of the dependence of the complex viscosity on the angular frequency of shear at 30 °C for the obtained oligoglycerols with the use of ionic liquids (**a**) Newtonian liquids (**b**) non-Newtonian liquids.

Additionally, the differences in the composition of glycerin oligomerization post-reaction mixtures (the presence of a cyclic dimer) obtained against ionic liquids with dodecyl substituents in the cation ([C_12_-tea][OAc], [C_12_-mim][OAc], [C_12_-mim][NaHPO_4_]) from products obtained with respect to other catalysts seem to confirm the results of their rheological tests. Obtained results of complex viscosity analysis |η*| of the obtained post-reaction mixtures as a function of the shear strain frequency [ω] are shown in Figure 9.

It turned out that all tested glycerol oligomerization products (Figure 9) were characterized by a higher complex viscosity in the analyzed shear strain range compared to pure glycerol, which proves their higher molecular weight. Thus, the obtained rheological relationships confirm the effectiveness of the obtained ionic liquids as catalysts for the synthesis of low-molecular-weight oligoglycerols. In addition, a different course of rheological curves was found for the post-reaction mixtures obtained for [C_12_-tea][OAc], [C_12_-mim][OAc], and [C_12_-mim] [NaHPO_4_] (Figure 9a), compared to the dependencies obtained for other mixtures (Figure 9b). The products obtained in the presence of imidazolium ionic liquids containing dodecyl substituents in the cation, regardless of the anion type, as well as of the ammonium liquid with the same substituent, behave like Newtonian liquids, for which the complex viscosity does not depend on the shear frequency. As was evident from the MS analysis, all these mixtures, apart from di- and triglycerol and unreacted glycerol, additionally contained α, α-cyclodiglycerol, the presence of which seems to reduce the viscosity of the composite sample, especially at higher values of the shear frequency. Among the three glycerol oligomerization products compared here, the mixture obtained with the ionic liquid [C_12_-tea][OAc] has a clearly higher value of complex viscosity in the entire range. This is probably due to the higher proportion of oligoglycerols in this mixture, as evidenced by the highest glycerin conversion value obtained for this liquid, i.e., 92% (Table 2). In contrast, mixtures of linear, low-molecular-weight glycerol oligomers (as evidenced by MS data) obtained in relation to the remaining ILs (Figure 9b) show the properties of non-Newtonian liquids, for which the complex viscosity depends on the shear frequency, especially at its higher values. For these mixtures of linear glycerin oligomers (dimer and trimer), a sharp increase in complex viscosity was observed with an increase in shear frequency, which may be due to the formation of intermolecular hydrogen bonds. Overall, the behavior of such post-reaction mixtures appears to be similar to those of dilatation fluids, which, with increasing shear frequency, change the properties from a viscous fluid to an elastic solid.

## 4. Conclusions

The studies on glycerin oligomerization described above show that the obtained ionic liquids undoubtedly show catalytic activity in the glycerin oligomerization reaction, similar to the homogeneous catalyst sodium carbonate. Moreover, for some of them, especially [C_12_-tea][OAc], the rate of reaction and the degree of conversion of the substrate (under the same conditions) clearly exceed the corresponding capacity, not only of other ionic liquids, but also of this known homogeneous catalyst. All tested catalysts, under the applied oligomerization conditions, lead to the preparation of low molecular weight oligoglycerols (mixtures of dimers and trimers).

## Figures and Tables

**Table 1:** Values of Hammett function (H_0_) of ionic liquids.

IL Type	A_max_	[I^−^] (%)	[HI] (%)	Hammett Function
				H_0_
[C_2_-mim][Br]	0.058	6.08	93.92	11.21
[C_2_-mim][OAc]	0.173	18.06	81.94	11.74
[C_12_-mim][OAc]	0.650	67.85	32.15	12.72
[C_14_-mim][OAc]	0.735	76.76	23.24	12.92
[C_12_-tea][OAc]	0.610	63.67	36.33	12.64
[C_12_-mim][NaHPO_4_]	0.495	51.69	48.31	12.43
[C_14_-mim][NaHPO_4_]	0.718	75.03	24.97	12.88

**Table 2:** The influence of the catalyst on the efficiency of glycerin oligomerization reaction (180 °C, 400 mbar, 3 h).

Catalyst	Reaction Product Symbol	Reaction Rate Constant *k* [s^−1^]	Regression Coefficient R^2^	Glycerols Conversion after 3 h [%]	Hammett Function H_0_
Na_2_CO_3_	PGL 1	1.56 × 10^−4^	0.991	86	-
[C_2_-mim][Br]	PGL 8	1.74 × 10^−4^	0.749	89	11.21
[C_2_-mim][OAc]	PGL 2	1.51 × 10^−4^	0.925	85	11.74
[C_12_-mim][OAc]	PGL 6	1.44 × 10^−4^	0.952	83	12.72
[C_14_-mim][OAc]	PGL 3	1.35 × 10^−4^	0.878	82	12.92
[C_12_-tea][OAc]	PGL 5	2.10 × 10^−4^	0.936	92	12.64
[C_12_-mim][NaHPO_4_]	PGL 7	1.49 × 10^−4^	0.971	85	12.43
[C_14_-mim][NaHPO_4_]	PGL 4	1.14 × 10^−4^	0.994	76	12.88
